# Indirect CT venography of the lower extremities: impact of scan delay and patient factors on contrast enhancement and examination quality

**DOI:** 10.1007/s00330-022-08841-0

**Published:** 2022-05-12

**Authors:** Thien Trung Tran, Cathrine Helgestad Kristiansen, Owen Thomas, Sumit Roy, Felix Haidl, Haseem Ashraf, Nils Einar Kløw, Knut Stavem, Peter M. Lauritzen

**Affiliations:** 1 Institute of Clinical Medicine, Faculty of Medicine, University of Oslo, Oslo, Norway; 2 Department of Diagnostic Imaging and Intervention, Akershus University Hospital, Lørenskog, Norway; 3 Department of Life Sciences and Health Radiography, Oslo Metropolitan University, Oslo, Norway; 4 Health Services Research Department (HØKH), Akershus University Hospital, Lørenskog, Norway; 5 Department of Anaesthesiology, Akershus University Hospital, Lørenskog, Norway; 6 Division of Radiology and Nuclear Medicine, Oslo University Hospital Ullevål, Oslo, Norway; 7 Department of Pulmonary Medicine, Akershus University Hospital, Lørenskog, Norway

**Keywords:** Deep vein thrombosis, Venography, Heart rate, Blood pressure, Cardiac output

## Abstract

**Objectives:**

Indirect computed tomography venography (CTV) is often the next imaging modality for deep vein thrombosis (DVT) when sonography is inconclusive. Our aim was to investigate the impact of scan delay and patient factors on contrast enhancement (CE) and examination quality in CTV.

**Methods:**

Patients with clinical suspicion or clinical mimics of DVT in one large hospital were enrolled. Age, sex, body weight, height, heart rate, systolic blood pressure and cardiac output were registered. CTV of the popliteal veins was obtained at 30 s intervals at 30–210 s delays. The proportions of examinations with CE exceeding predefined cut-offs were estimated and subjective examination quality was rated. Changes in CE with time, and associations between patient factors and time to peak contrast enhancement (TPCE) were modelled with mixed effects non-linear and linear regression, respectively.

**Results:**

The CE increased with increasing scan delay and reached a plateau from 120 to 210 s. The percentages of examinations achieving enhancement above cut-offs across all thresholds from 70 to 100 HU were higher at 120 s compared to 90 s (*p* < 0.001). After 120 s, there were no differences across scan delays for any thresholds. No patient factors showed a significant effect on TPCE. The percentage of examinations rated as acceptable was higher at 120 s compared to 90 s (*p* < 0.001). After 120 s, there were no statistically significant differences across scan delays.

**Conclusions:**

No patient factors were associated with TPCE in CTV. A fixed scan delay of 120–210 s yielded the best examination quality.

**Key Points:**

*• Contrast enhancement reached a plateau at scan delay between 90 and 120 s.*

*• A scan delay of 120–210 s yielded the best examination quality.*

*• No patient factors were associated with time to peak contrast enhancement.*

## Background

Venous thromboembolism (VTE) encompasses deep vein thrombosis (DVT) and pulmonary embolism. VTE is common and occurs with an incidence of up to 200 per 100,000 person-years [1, 2]. Common risk factors for VTE are increasing age, obesity, major trauma, active cancer and pregnancy [1]. Emboli originating from lower extremity DVT (LE-DVT) is a common cause of pulmonary embolism. Massive pulmonary embolism may lead to pulmonary hypertension, hemodynamic collapse or death [3].

Sonography is the preferred initial diagnostic tool to diagnose LE-DVT. However, the modality has limited visualization of deeper structures such as pelvic veins and inferior vena cava as well as in obese patients [3–5]. Alternative methods are indirect computed tomography venography (CTV), magnetic resonance venography (MRV) and conventional venography (CV). MRV shows soft tissue very well and in some instances better than CTV [6, 7]. CTV and MRV are both superior to sonography for the detection of differential diagnoses of DVT, such as muscular hematoma, abscess, popliteal cyst or compartment syndrome [7–11]. In addition, indirect CTV and MRV are helpful for planning pharmaco-mechanical thrombectomy, enabling visualisation of the proximal and distal thrombus extension [12, 13]. Although MRV has the advantage of requiring no contrast administration and implying no radiation exposure, it is less available, costlier and more time-consuming [14]. Hence, in many centres, CTV is the next investigation of choice that may add information when sonography is not diagnostic.

When performing indirect CTV, the contrast medium (CM) is usually injected into the antecubital vein. Conversely, both direct CTV and CV have the CM injected peripherally into the involved extremity. Direct CTV and CV use the first pass contrast enhancement (CE) for evaluation while indirect CTV scans the delayed phase when opacification of the veins has reached steady-state after 60–120 s or more from the start of CM injection [15, 16]. Due to a wide time window of the venous steady-state, ranging from 60 s after CM injection to more than 420 s, the practice varies between hospitals when using a fixed scan delay [16–21].

Most thrombi have an attenuation ranging from 30 to 70 Hounsfield units (HU), where erythrocyte-rich and acute thrombi have higher densities than platelet-rich and chronic thrombi [16, 22–25]. Therefore, CE with venous attenuation above 70 HU is usually considered adequate for the diagnosis of DVT [26, 27].

Several studies have reported an association between patient factors and the time to peak contrast enhancement (TPCE) in the arterial and the hepatic phase [28]. There are some reports of associations between patient factors and venous CE in the lower extremities at a fixed scan delay, but to our knowledge, the relationship between patient factors and TPCE is yet to be determined [20, 21].

The aim of this study was to investigate the impact of scan delay on CE and examination quality of popliteal veins. We also aimed to investigate if patient factors, namely: age, sex, body weight, systolic blood pressure, heart rate, cardiac output (CO) and DVT may influence the TPCE.

## Methods

### Ethical approval

The study was approved by the Regional Committee for medical and health research ethics of South-Eastern Norway and Akershus University Hospital data protection officer. Written informed consent was obtained from all patients. The study was not registered at clinicaltrials.gov.

### Patient population

**Fig. 1:**
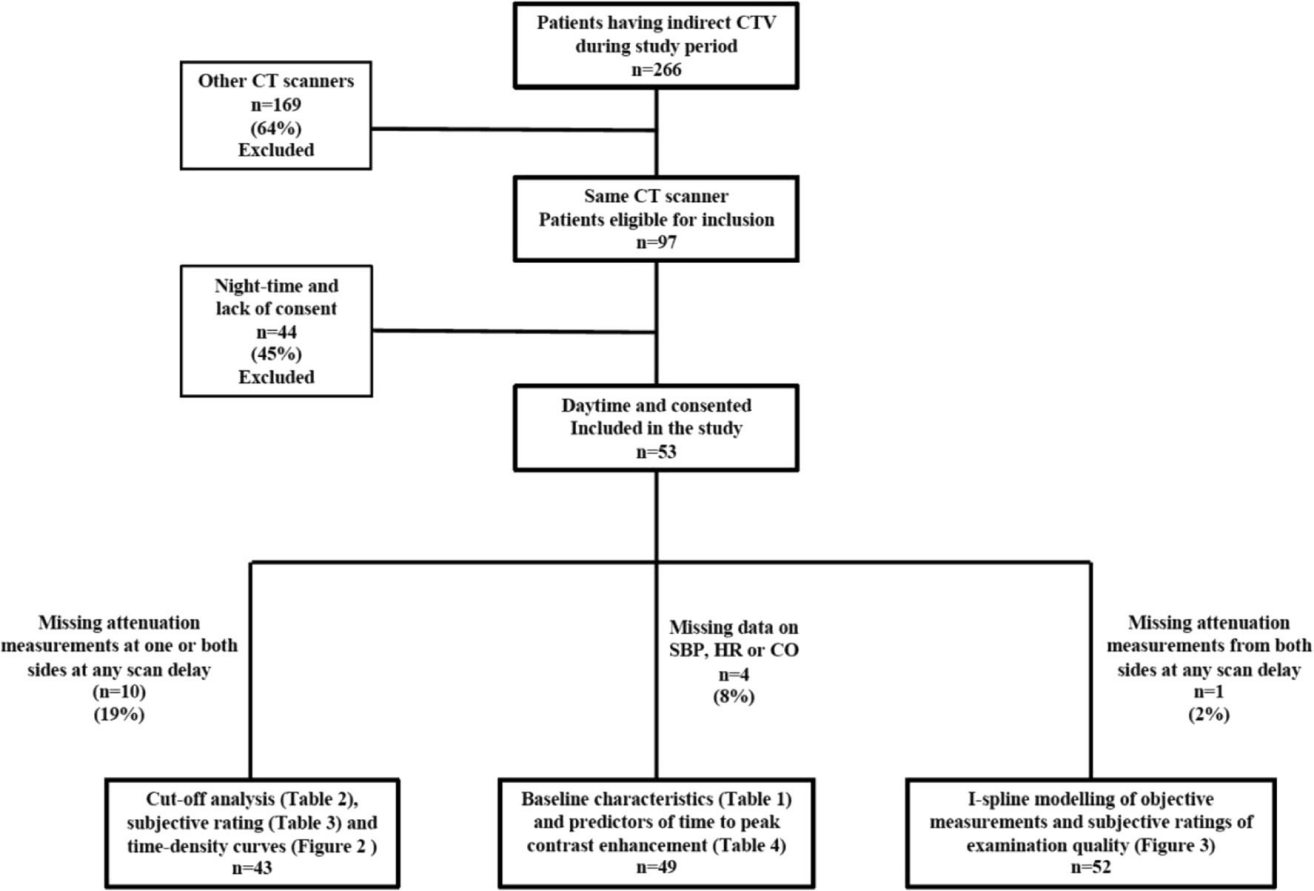
Flow chart of patients eligible for indirect lower limb CTV during the study period and the % excluded with the reason of exclusion

Between 2017 and 2020, patients with clinical suspicion of DVT and clinical mimics of DVT were prospectively enrolled in this study. Exclusion criteria were age < 18 years, pregnancy, previous allergic reaction to CM and renal impairment. A flowchart of patients eligible for indirect lower limb CTV during the study period and the % excluded with the reason of exclusion is shown in Fig. 1.

### Indirect CT venography procedure

#### Contrast medium injection

The intravenous CM, iopamidol 350 mgI/mL (Iomeron 350H, Bracco) or iohexol 350 mgI/mL (Omnipaque 350H, GE Healthcare), was administered using a power injector (CT Exprès TM 4D, Bracco Injeneering S.A.) through the antecubital vein followed by a saline flush.

CM dose was estimated based on the perceived body composition of the patients, rated as obese, average, or muscular. Obese patients received an approximation of 1.3 mL/kg, average 1.8 mL/kg and muscular 2.3 mL/kg. According to the standard protocol, the total amount of administered CM ranged from 90 to 180 mL.

#### CT acquisition parameters

All CT scans were performed on a 256-slice multi-detector CT scanner (Brilliance iCT; Philips Healthcare).

The main scan covered the region from the diaphragm to the feet and was started 120 s after the start of CM injection with a fixed CM injection time of 40s and a fixed post-CM injection delay of 80s. In addition, a single acquisition was taken at the level of the proximal popliteal veins at a fixed scan delay of 30 s, 60 s, 90 s, 150 s, 180 s and 210 s after the start of CM injection.

The tube voltage was chosen based on the patient’s body mass index (BMI) to achieve highest possible vessel enhancement without compromising image quality. At the level of popliteal veins, 80 kilovolt (kV) was used for patients with BMI ≤ 25 and 100 kV for patients with BMI > 25.

#### Patient monitoring

During contrast administration and scanning, hemodynamic data (heart rate, blood pressure and CO) were measured continuously and non-invasively by Nexfin HD monitor (BMEYE), a photo-plethysmography device using an inflatable finger cuff as the only interface with the patient [29, 30]. Means of recorded samples (4 min) covering from before the start of CM injection to after completed scanning were used in the analyses.

#### Patient variables

Data on the patients’ age, sex, body weight and self-reported height were collected.

BMI was calculated as (body weight in kg) / (height in m)^2^.

### Image reconstructions and evaluations

#### Image reconstructions

Conventional images were reformatted to a slice thickness of 3 mm. All images were sent to a commercial workstation (IntelliSpace Portal 10, Philips Healthcare) and a Carestream Vue picture archiving and communication system version 12 (Carestream Health) for evaluation.

#### Objective measurements of examination quality

CE was measured in the popliteal veins bilaterally by placing a circular region of interest cursor on the single acquisitions taken at 30 s, 60 s, 90 s, 150 s, 180 s, 210 s and from the full acquisition scan taken at 120 s, and registering the attenuation values (in Hounsfield units). The circular regions of interest were drawn as large as possible within the anatomic limits of the vessel lumen, avoiding artefacts. The CE measurements were performed by a radiographer with more than 10 years of experience in CT cardiovascular imaging and served as the objective measurement of examination quality.

#### Subjective rating of examination quality

The subjective rating was performed independently by two cardiovascular interventional radiologists with more than 10 years’ experience. Examinations were rated at each scan delay from 30 to 210 s, and the raters were not blinded to the scan delay.

Examinations were rated adequate if opacification of the popliteal veins and image quality were sufficient for detecting or ruling out DVT; otherwise, they were rated inadequate. Disagreement between the two radiologists was independently rated by a third radiologist and resolved by the median score for all three radiologists.

### Statistical analysis

Descriptive statistics are reported using mean (SD) or number (%), as appropriate. The time-density curves for the popliteal veins were illustrated using a boxplot (median, 25% and 75% percentiles).

Objective evaluation of popliteal CE was performed by estimating the proportion of examinations exceeding predefined cut-offs of 70, 80, 90 and 100 HU at scan delays between 90 and 210 s. For standardisation, only patients with a complete set of measurements at scan delays between 90 and 210 s were included.

Differences between scan delays in proportions exceeding the cut-offs were tested with a logistic mixed model trained on binary endpoints with patient identity as a random effect and time as a fixed effect. The binary endpoint was whether the popliteal CE exceeded the given threshold.

We also estimated the peak CE defined as the highest attenuation value for each patient, and the TPCE defined as the scan delay at which the highest attenuation was achieved.

The subjective rating of examination quality according to scan delay was reported as the percentage of adequate examinations between 90 and 210 s. The inter-rater agreement (%) and Cohen’s kappa score were estimated [31]. The strength of agreement was rated as poor, slight, fair, moderate, substantial or almost perfect according to the Kappa statistic scores < 0.00, 0.00–0.20, 0.21–0.40, 0.41–0.60, 0.61–0.80 and 0.81–1.00 respectively [32]. Differences between scan delays in the proportions of adequate examinations were tested with a logistic mixed model trained on the subjective ratings by the raters, using patient and rater identity as random effects and time as a fixed effect.

A Bayesian mixed-effects non-linear spline regression model was used to model the variation of CE and subjective rating of examination quality with time [33]. We used fixed effects in the form of an I-spline basis of degree three and knots at 90 s and 150 s, and random effects of a constant and linear term for each individual and a constant term for rater identity for subjective ratings [34]. The I-spline fixed effects provide a basis for six monotonic functions that can capture the global non-linearity in the relationship between the outcome variable and time, while the random effects allow for variation in scale between individuals and raters. The I-spline basis is shown in Fig. 3a. A Gaussian likelihood was used for the CE measurements and a logistic likelihood for the binary subjective ratings.

The trained mixed-effects spline model allows for estimation of the time at which the latent variables underlying the models reach a plateau: for the Gaussian likelihood, this can be interpreted as a smoothed value of the contrast enhancement, while for the logistic likelihood the latent variable can be interpreted as the log odds ratio of the rating being judged acceptable.

For both CE and subjective rating, we defined the plateau threshold as the maximum of the model latent variable mean across scan delays minus half the predicted standard deviation, with the prediction including all fixed and random effects. The time until the plateau was reached was defined as the minimum time point at which the predicted mean value is greater than the plateau threshold.

A linear mixed-effects model was used to evaluate associations between patient factors and the TPCE. A significance level of 0.05 was chosen for the analysis.

Patients with missing data on heart rate (6%), systolic blood pressure (8%) or CO (6%) were excluded from the analyses of baseline characteristics and predictors of TPCE.

Patients with missing attenuation measurements (19%) (due to thrombus at one or both sides (*n* = 3) or missing image acquisitions at any scan delay (*n* = 7)) were excluded from the cut-off analyses to archive a complete set of data for comparison.

Statistical analyses were performed with R version 4.1.0 (R Foundation for Statistical Computing) using the libraries brms and lme4, or Stata version 15.1 (StataCorp) [35].

## Results

Fifty-three patients were included in the study. The mean age was 56 years. Thirty-five patients (66 %) were male. Data on patient characteristics are summarized in Table 1. Thirty-six patients (68%) were referred due to suspicion of LE-DVT, 24 (45%) on acute and 12 (23%) on chronic basis. In total, 13 patients (25%) had verified LE-DVT.

**Table 1:** Baseline patient characteristics, mean (SD)

Age (years)	56 (13)
Male sex, number (%)	35 (66)
Body weight (kg)	90 (22)
Body mass index (kg/m^2^)	28.4 (6.0)
* Systolic blood pressure (mmHg)	131 (21)
* Heart rate (beats/min)	78 (11)
* Cardiac output (L/min)	8.3 (2.1)

*n* = 53

*Four patients with missing data on systolic blood pressure, heart rate or cardiac output were excluded

### Objective measurements of examination quality

**Fig. 2:**
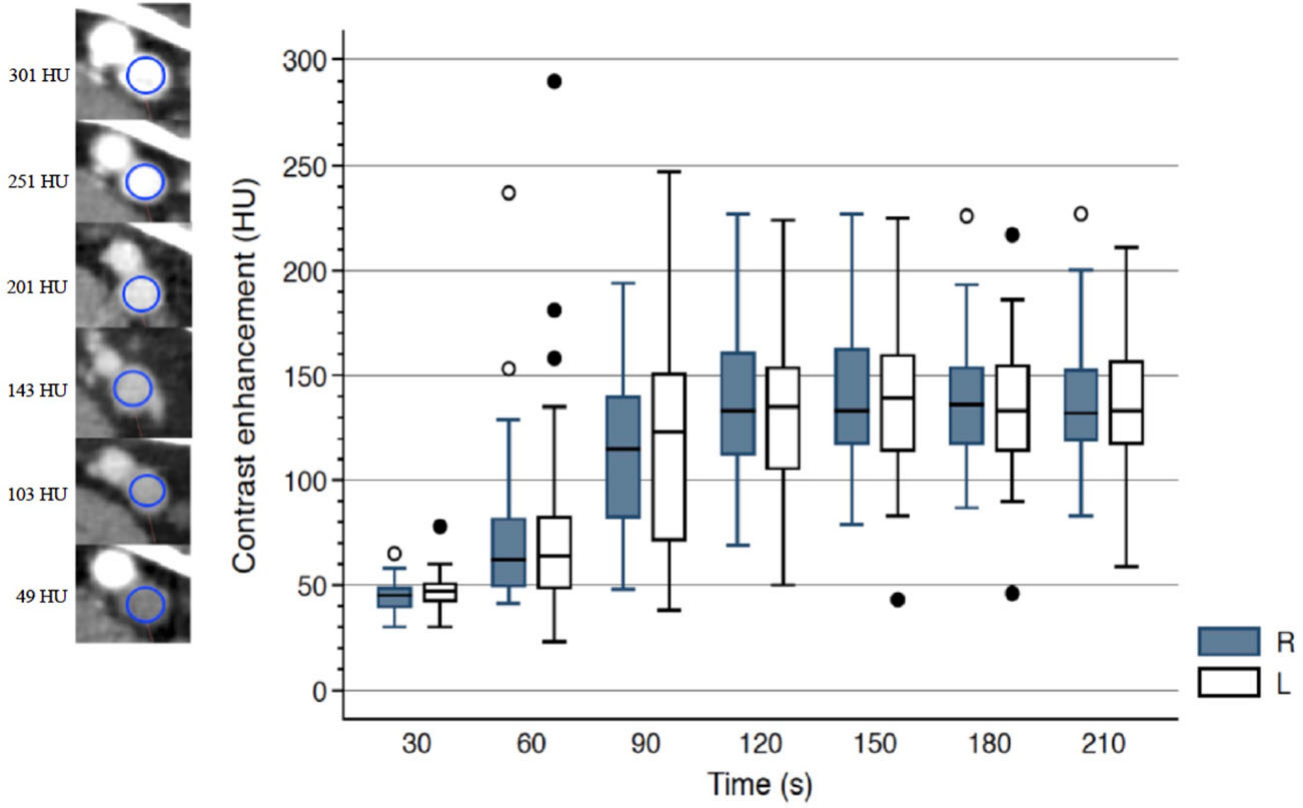
Time-density curves of popliteal venous contrast enhancement vs. scan delay (s) after contrast injection. The box shows the 25^th^ to 75^th^ percentile. The horizontal bar inside the box shows the median value. The upper and lower bars show the upper and lower adjacent values (values within 1.5 times the interquartile range (IQR) from the upper and lower quartile). Outer values are shown as dots and circles. The IQR is defined as the distance between the 25^th^ and 75^th^ percentile. Images to the left for the Y-axis demonstrate the placement of regions of interests in the popliteal veins, illustrating different densities between 50 HU and 300 HU. (*n* = 43). 10 patients were excluded due to missing data on one or both sides at a given time point. R right side, L left side, HU Hounsfield unit

The CE increased with increasing scan delay after CM injection and reached a plateau from 120 to 210 s (Fig. 2).

**Fig. 3:**
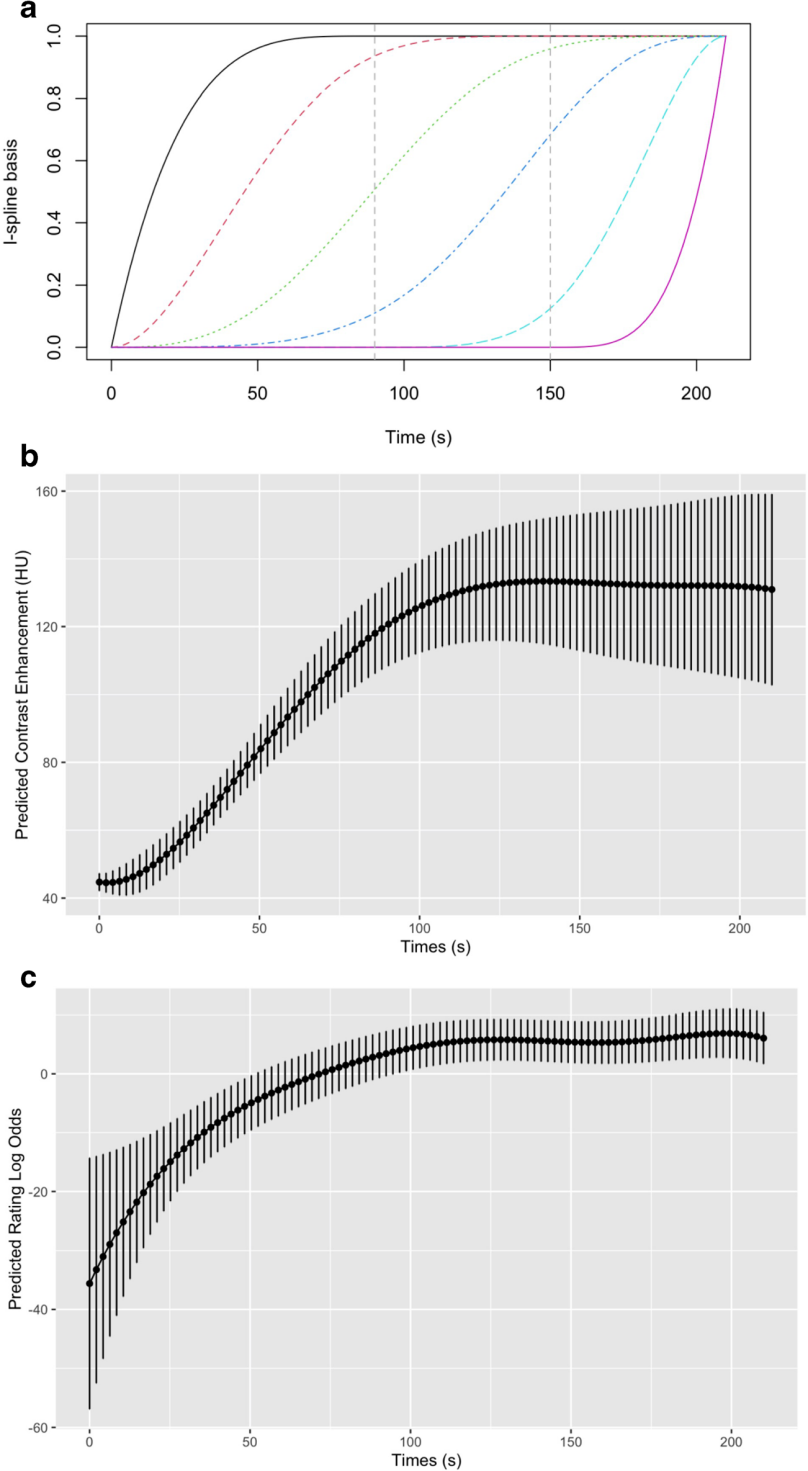
**a.** An I-spline basis with knots at *t* = 90 s and *t* = 150 s and a degree of 3, used to model the non-linear relationship between contrast enhancement and time and subjective ratings of examination quality (n = 52). One patient was excluded due to missing data from both sides at a given time point. The analysis was based on the average between the sides. If missing data on one side, the average is taken to be the value of the observed side. **b** I-spline modelling of objective measurements of examination quality, representing the variation of the prediction of contrast enhancement (HU) with time alongside the associated uncertainty. For locating the plateau, we find the maximum of the predicted mean (found at 140.7 s), then use the value of the mean with half of the associated standard deviation subtracted as the value of the plateau—approximately 125 HU. The minimum predicted mean value which reached the plateau value was 97 s: we interpret this to be the point at which the curve reaches a plateau. **c** I-spline modelling of subjective ratings of examination quality, modelling the log odds ratio of a rater giving a subjective positive rating with time. We use the maximum mean value minus half the standard deviation to define the plateau value. The maximum location is at 197.4 s, and the plateau value is 4.8. The lowest time at which the plateau value is achieved by the mean is 105 s, which we can interpret as being the point at which the plateau is reached

The mixed-effects model using the I-spline basis was fitted successfully, with the basis shown in Fig. 3a and CE model predictions in Fig. 3b. For the CE models, the plateau was reached between scan delays of 90 s and 120 s (at 97 s). At this point, the median predicted plateau height was 122 HU, with first and third quartiles of 115 HU and 132 HU, incorporating the variation between individuals and epistemic uncertainty.

The percentages of examinations achieving enhancement above cut-offs across all thresholds from 70 to 100 HU were significantly higher at 90 s compared to 60 s and at 120 s compared to 90 s. After 120 s, there were no statistically significant differences across scan delays for any thresholds (Table 2). From this, we might reasonably claim that a plateau is reached somewhere between 90 and 120 s.

**Table 2:** Popliteal venous contrast enhancement above selected cut-offs according to scan delay (s) after contrast injection

Cut-off level	Scan delays														
	90 s			120 s			150 s			180 s			210 s		
	*p*-value*	R	L	*p* value*	R	L	*p* value*	R	L	*p* value*	R	L	*p* value*	R	L
> 70 HU	< 0.001	35 (81)	33 (77)	< 0.001	42 (98)	41 (95)	0.16	43 (100)	42 (98)	0.99	43 (100)	42 (98)	0.99	43 (100)	42 (98)
> 80 HU	< 0.001	33 (77)	30 (70)	< 0.001	41 (95)	40 (93)	0.10	42 (98)	42 (98)	0.43	43 (100)	42 (98)	0.43	43 (100)	42 (98)
> 90 HU	< 0.001	31 (72)	29 (67)	< 0.001	39 (91)	38 (88)	0.20	42 (98)	41 (95)	0.99	42 (98)	41 (95)	0.53	42 (98)	42 (98)
> 100 HU	< 0.001	28 (65)	28 (65)	< 0.001	36 (84)	33 (77)	0.14	40 (93)	37 (86)	0.33	41 (95)	39 (91)	0.43	41 (95)	41 (95)

Numbers are *n* (%)

*n* = 43

10 patients were excluded due to missing data on one or both sides at a given time point

*R* right side, *L* left side, *HU* Hounsfield unit

*The *p* values are associated with effects in the named intervals compared to the preceding scan delay (i.e. *p* values at 90 s represent the difference between 60 and 90 s). The *p* values are derived from a logistic mixed model trained on binary endpoints. Patient identity was used as a random effect and time as a fixed effect, the binary endpoint was whether the contrast enhancement had achieved the given threshold

Mean TPCE was 157 s (SD 45), and mean peak CE was 157 HU (SD 36). In the linear mixed-effects model including both popliteal veins, no patient factors showed a significant effect on TPCE (Table 3).

**Table 3:** Predictors of time (s) to peak contrast enhancement in popliteal veins, univariate linear mixed-effects model

	Unstandardized beta	95% CI	*p*
Age, per 10 years	4.56	−3.07, 12.19	0.242
Male sex	−5.90	−26.01, 14.20	0.565
Body weight, kg	−0.15	−0.60, 0.29	0.503
Systolic blood pressure, per 10 mmHg	−0.45	−5.05, 4.15	0.849
Heart rate, per 10 beats/min	−7.38	−15.85, 1.09	0.088
Cardiac output (L/min)	−3.46	−8.27, 1.35	0.159
Deep vein thrombosis	20.59	−5.10, 46.28	0.116

*n* = 98 observations, 49 patients

*CI* confidence interval

Four patients with missing data on systolic blood pressure, heart rate or cardiac output were excluded

### Subjective rating of examination quality

The mixed-effects model using the I-spline basis was fitted successfully, with the spline basis shown in Fig. 3a and subjective rating model log odds ratio predictions in Fig. 3c.

The model showed that the subjective examination quality reached a plateau between scan delays of 90 and 120 s (at 105 s).

The percentage of examinations rated as acceptable was significantly higher at 120 s compared to 90 s. After 120 s, there were no statistically significant differences across scan delays (Table 4).

**Table 4:** Subjective rating of examination quality at the level of the proximal popliteal vein, according to scan delay (s) after contrast injection; scores and agreements between raters

	90 s		120 s		150 s		180 s		210 s	
	R	L	R	L	R	L	R	L	R	L
Rater 1	29 (67)	26 (60)	42 (98)	41 (95)	43 (100)	42 (98)	43 (100)	42 (98)	41 (95)	42 (98)
Rater 2	22 (51)	21 (49)	37 (86)	35 (79)	37 (86)	35 (81)	36 (84)	37 (86)	32 (74)	35 (81)
*p* value*	< 0.001		< 0.001		0.40		0.37		0.10	
% agreement **	83.7	83.7	83.7	86.1	86.1	83.7	83.7	88.4	73.8	83.3
Kappa **	0.67	0.68	−0.04	0.35	0	0.19	0	0.26	−0.05	0
Mean kappa***	0.67		0.16		0.09		0.13		−0.02	
Resolved rating ****	25 (58)	24 (56)	38 (88)	38 (88)	40 (93)	38 (88)	40 (93)	39 (91)	39 (91)	38 (88)

Numbers are *n* (%)

*n* = 43

10 patients were excluded due to missing data on one or both sides at a given time point

*R* right side, *L* left side

*p values associated with time intervals, derived from a logistic mixed model trained on the subjective ratings by the rates, using patient and rater identity as random effects and time as a fixed effect. The *p* values represent the difference between the named and the preceding scan delay (i.e. *p* values at 90 s represent the difference between 90 and 60 s)

**Agreement and kappa between rater 1 and rater 2

***Mean of kappa for left and right side

****Disagreements between raters 1 and 2 were independently assessed by rater 3 and resolved by median score for all three raters

The inter-observer agreement was consistently above 80% with one exception for scan delays between 90 and 210 s (Table 4). The kappa was substantial (0.67) at 90 s scan delay, but poor-slight (between −0.52 and 0.35) at scan delays between 120 and 210 s.

## Discussion

In this study, we found that objective and subjective measurements of image quality reached a plateau between 90 and 120 s after CM injection, and no differences were found at scan delays beyond 120 s.

### Objective measurements of examination quality

Our time-density curves showed that CE increased with time until a plateau was reached from 120 to 210 s after CM injection, which corresponds with the findings of Szapiro et al [16]. The CE variation between 120 and 210 s was small and I-spline modelling indicated that this variation of CE was within the range of noise and that a peak CE could not be identified within the plateau.

According to previous studies, CE above 70 HU in the popliteal veins is sufficient to distinguish a thrombus from surrounding CE and soft tissues, since most thrombi are below 70 HU [16, 22–25]. Hence, 70 HU, 80 HU, 90 HU and 100 HU were chosen as cut-offs in our subgroup analyses. We found that the percentage of examinations achieving CE above cut-off was higher at 120 s compared to 90 s, but after 120 s, there were no differences across scan delays for any thresholds, which is consistent with the I-spline model.

In the linear mixed-effect model, no patient factors were associated with TPCE. Hence, we found no evidence to support tailoring scan delay to age, sex, body weight, systolic blood pressure, heart rate, CO or presence of DVT.

### Subjective rating of examination quality

The results of the subjective rating of examination quality were consistent with the objective measurements. However, differences between raters suggest that other factors in addition to venous CE were addressed. The inter-observer agreement was consistently above 80% with one exception, but low kappa values between −0.52 and 0.35 were seen at scan delays 120 to 210 s. The kappa calculation is considered a more robust measure than just measuring the proportion of agreements since kappa adjusts for agreements occurring by chance. However, in this case, the proportions of inadequate examinations were low resulting in a high probability of agreement by chance. In cases like ours where the distribution of ratings between categories is highly skewed, the kappa may be paradoxically low even though %-agreement is high. In this situation, the expected agreement may be high, close to the observed agreement, explaining a low kappa [36, 37]. Furthermore, as the raters were not blinded to the scan delay, a rater with a preference for a specific scan delay may have biased the results.

### Strengths and weaknesses

The strength of the present study is that data was collected prospectively and it addresses an almost 20-year-old topic to a newer CT scanner technology. Both objective and subjective measurements of quality were analysed. In addition, data on patient factors were collected to analyse possible associations with TPCE to evaluate if a patient-tailored scan delay may be preferable to a fixed scan delay.

There are several limitations in our study. All our imaging data were acquired at the level of the proximal popliteal veins which may limit the validity of our results at the more proximal and distal anatomical levels. Also, we only included a limited number of patients that consented and that could be examined during daytime. Furthermore, patients with missing data on patient factors and missing imaging data were excluded from the analyses as part of standardization, making the sample size smaller. This weakened the statistical strength when analysing possible associations between patient factors and TPCE. Finally, the Nexfin device used to measure CO is non-invasive and has been reported to be a valid method compared to thermodilution, FloTrac/vigileo and echocardiography [29, 30, 38]. However, others have questioned the validity of the same device in certain groups of patients [39–41]. The baseline values of heart rate and CO were relatively high for patients at rest in our study, which may be explained by the stress associated with the examination, but this might also be explained by measurement uncertainties related to the Nexfin. The strength of the Nexfin measurement lies in its ability to detect variations accurately, such as in follow up of patients, but may be less accurate as an absolute value measurement [42].

In conclusion, we found that no patient factors were associated with the TPCE in CTV and that a scan delay of 120–210 s yielded the best examination quality.

## Abbreviations

CE: Contrast enhancement
CM: Contrast medium
CO: Cardiac output
CTV: CT venography
DVT: Deep vein thrombosis
LE-DVT: Lower extremity DVT
TPCE: Time to peak contrast enhancement
VTE: Venous thromboembolism

## Declarations

### Guarantor

The scientific guarantor of this publication is Peter M. Lauritzen.

### Statistics and biometry

One of the authors has significant statistical expertise.

### Methodology

• prospective

• diagnostic/experimental

• performed at one institution
